# CD19^+^CD11c^+^T-bet^+^ B cells in myasthenia gravis: a potential biomarker

**DOI:** 10.3389/fneur.2025.1623066

**Published:** 2025-08-22

**Authors:** Yaru Lu, Huimin Shen, Yiye Wang, Kai Ma, Ruihua Sun, Ying Zhao, Yaqiong Li, Qian Ma, Jiewen Zhang

**Affiliations:** ^1^Department of Neurology, Zhengzhou University People’s Hospital, Henan Provincial People’s Hospital, Zhengzhou, China; ^2^Department of Neurology, The Fifth Affiliated Hospital, Sun Yat-sen University, Zhuhai, China

**Keywords:** myasthenia gravis, NF-κB pathway, age-associated B cells, proteomics, flow cytometry

## Abstract

**Background:**

Myasthenia gravis (MG), an autoimmune disorder characterized by B cell-driven autoantibody production, exhibits heterogeneous B cell subsets dysregulation and incompletely defined signaling mechanisms.

**Methods:**

A cohort of 20 naïve MG patients positive for anti-acetylcholine receptor (AChR) antibodies and 15 healthy controls was analyzed. Peripheral blood mononuclear cells underwent proteomic profiling, flow cytometry (age-associated B cells (ABCs), plasma cells, T follicular helper cells, and regulatory B cells), and western blot validation of nuclear factor kappa-B (NF-κB)/cellular reticuloendotheliosis oncogene homolog (c-Rel) expression. Clinical severity was assessed using quantitative MG (QMG) scores. Statistical analyses included differential protein expression, pathway enrichment, and receiver operating characteristic (ROC) curve evaluation.

**Results:**

Proteomics revealed significant activation of the B cell receptor and NF-κB/c-Rel signaling pathways in MG patients, validated by upregulated NF-κB/c-Rel expression (*p* < 0.01). Flow cytometry demonstrated elevated ABCs (CD19^+^CD11c^+^T-bet^+^), plasma cells, and T follicular helper cells, alongside reduced regulatory B cells in MG (*p* < 0.001). The proportion of ABCs correlated positively with QMG scores (*r* = 0.5015, *p* = 0.024) but not with AChR antibody titers, suggesting antibody-independent mechanisms. ROC analysis identified moderate diagnostic utility of ABCs for moderate-to-severe MG (QMG scores ≥ 6; area under the curve = 0.68, 95% confidence intervals: 0.42–0.94).

**Conclusion:**

This study establishes ABCs and NF-κB/c-Rel signaling as central contributors to AChR-MG immunopathology. Therefore, ABCs may serve as complementary biomarkers for clinical stratification.

## Highlights

- B cell pathway activates and B-cell subsets imbalance in AChR-MG.
- ABCs are positively correlated with the disease severity in AChR-MG.
- ABCs could serve as a potential biomarker for AChR-MG.

## 1 Introduction

Myasthenia Gravis (MG) is an autoimmune disorder characterized by autoantibodies targeting postsynaptic neuromuscular junction proteins, leading to muscle weakness and fatigue (1, 2). Its clinical phenotype is influenced by multiple factors (3). B cell dysregulation, aberrant T follicular helper (Tfh) cell activity, and the production of pathogenic autoantibodies against specific targets are central to MG pathogenesis (4). Despite therapeutic advances, including immunosuppressants and B cell-targeted biologics such as rituximab, a significant subset of patient’s exhibit treatment resistance or relapse (5–7), underscoring an incomplete understanding of the underlying immune mechanisms driving B cell pathology.

A critical unresolved aspect is the role of specific, recently characterized B-cell subsets in MG. Age-associated B cells (ABCs, CD19^+^T-bet^+^CD11c^+^) have emerged as pivotal players in autoimmune diseases such as systemic lupus erythematosus and rheumatoid arthritis, where they correlate with disease activity and contribute to pathogenesis through autoantibody production and pro-inflammatory cytokine secretion (8–15). ABCs also exhibit plasticity and context-dependent functions, including potential anti-inflammatory effects (16). Crucially, the expansion of ABCs has been validated as a biomarker of autoimmune conditions (11–15). However, the dynamics, clinical relevance, and potential biomarkers of ABCs in MG remain unexplored.

ABC differentiation is influenced by pathways implicated in MG immune dysregulation. Notably, Tfh cells and nuclear factor kappa-B (NF-κB) signaling, both of which are altered in MG, play key roles in ABC generation and function (17–23). In particular, aberrant NF-κB activation, especially the non-canonical pathway, has been observed in MG B cells and promotes their activation and differentiation (24, 25). Furthermore, transcription factors such as zinc finger E-box binding homeobox 2, which regulate ABCs via Toll-like receptor 7/9 signaling (21–23), present potential common regulatory nodes. The specific role of these pathways in shaping the ABC compartment within the autoimmune milieu of MG is unknown. Given the established association between ABCs and disease activity in other antibody-driven autoimmune disorders and their plausible link to dysregulated pathways in MG, investigating ABCs offers a promising opportunity to identify novel biomarkers for improved patient stratification and management.

We hypothesized that ABCs contribute to disease severity in MG patients positive for anti-acetylcholine receptor (AChR) antibodies and may serve as a clinically relevant biomarker. Therefore, the primary goal of this study was to comprehensively characterize ABCs in MG patients using integrated proteomic and flow cytometry-based phenotypic analyses, and to evaluate their association with key clinical parameters to assess their potential as clinical biomarkers.

## 2 Patients and methods

### 2.1 Patients

A total of 20 MG patients and 15 healthy controls (HC) were included in this study, and the sex and age of the two groups were matched. All patients were newly diagnosed with generalized MG (gMG) positive for anti-AChR antibodies or had discontinued immunosuppressive medications (including steroids and immunosuppressants) for at last 3 months (defined as naïve MG) and met the following inclusion criteria: (1) diagnosed with gMG; (2) aged from 18 to 55 years old; (3) anti-AChR antibodies positive; (4) quantitative myasthenia gravis (QMG) scores ≥ 3 at screening; (5) no steroids or immunosuppressant treatments or withdrawal for at last 3 months; (6) no prior treatment with monoclonal antibodies or other biologics; and (7) thymectomy-free or surgery performed > 6 months prior; no thymoma history. Participants were excluded based on the following criteria: (1) acute infection within 2 weeks; (2) chronic infections such as tuberculosis and hepatitis B. (3) concurrent autoimmune diseases; (4) sever organ dysfunction (cardiac, pulmonary, hepatic, or renal) or malignancy; and (5) major cerebrovascular or cardiovascular disease. The QMG scores of all enrolled MG patients were independently evaluated and averaged by two specialized physicians. The gMG patients were classified according to the Myasthenia Gravis Foundation of America (MGFA) clinical classification criteria (types II-IV). AChR antibody titers were measured via radioimmunoprecipitation assay with a positivity threshold of ≥ 0.45 nmol/L. Additionally, the participants’ sex and age were recorded routinely. All the participants provided informed consent for inclusion in the study. All study protocols were approved by the Ethics Committee and Institutional Review Board of the Henan Provincial People’s Hospital.

### 2.2 Methods

#### 2.2.1 Peripheral blood mononuclear cells separation

Peripheral blood from each participant was collected into EDTA anticoagulant tubes. PBMCs were isolated from whole blood by Ficoll-Paque density gradient centrifugation (GE Healthcare, Uppsala, Sweden) following the methods mentioned in previous study (26).

#### 2.2.2 Proteinomics analysis

PBMCs from 10 MG patients and 6 HC were lysed using radio immunoprecipitation assay (RIPA) lysis buffer supplemented with protease and phosphatase inhibitors. Protein extracts were digested with trypsin (Promega, Cat# V5071) and analyzed by liquid chromatography–tandem mass spectrometry (LC–MS/MS). MS data were processed using Proteome Discoverer (version 2.4) with the UniProt human database. Proteins with >50% missing values were excluded from the expression matrix. The experimental parameters were provided in the Supplementary materials.

#### 2.2.3 Flow cytometry analysis

PBMCs (2 × 10^6^/mL) were plated in U-bottom 96-well plates (100 μL/well), washed twice with phosphate buffered saline (PBS, 2% FBS, 0.4% EDTA, 0.02% sodium azide), and stained with fixable viability dyes and surface antibodies (Biolegend, anti-human-CD19-APC, CD11c-Percp/Cy5.5, CD27-PE/Cy7, CD38-PE, CD4-PE/Cy7, CXCR5-FITC, PD1-APC) for 20 min at room temperature. After washing, a subset of cells was resuspended in transcription factor staining buffer and incubated with anti-human T-bet-APC or IL-10-PE antibodies for 45 min (light-protected). Cells were washed three times with PBS, resuspended in 200 μL PBS, and analyzed by flow cytometry (Cytoflex S, Beckman, United States) to determine subpopulation frequencies. The gating strategy was shown in the Supplementary Figure S1.

#### 2.2.4 Protein extraction and western blot analysis

Cellular proteins were lysed using RIPA lysis buffer (Beyotime, Cat# P0040) supplemented with protease and phosphatase inhibitors. Protein concentrations were determined by BCA assay (Thermo Fisher Scientific, Cat# 23227). Equal amounts of protein (20 μg) were separated on 7.5% SDS-PAGE gels and transferred to PVDF membranes (0.22 μm, Merck Millipore). After blocking with 5% skim milk or BSA for 1 h at room temperature, membranes were incubated overnight at 4°C with primary antibodies: NF-κB (Proteintech, Cat# 10409-2-AP), cellular reticuloendotheliosis oncogene homolog (c-Rel) (Proteintech, Cat# 67747-1-Ig), and *β*-actin (Beyotime, Cat# AF5003). Membranes were then incubated with HRP-conjugated secondary antibody (Beyotime, Cat# A0208) for 1 h at room temperature. Protein bands were visualized using chemiluminescent substrate (MedChemExpress, Cat# HY-K2005) on Bio-Rad ChemiDoc XRS + Imaging System. Quantification was performed with ImageJ software using *β*-actin as loading control.

#### 2.2.5 Statistical analysis

Data were analyzed using GraphPad Prism 8.0 and R Studio (v4.4.3). Differentially expressed proteins (DEPs) were identified using the following thresholds: absolute log2 (fold change) > 1 and Benjamini-Hochberg adjusted *p*-value <0.05 (limma package). DEPs were subjected to functional enrichment analysis in the Gene Ontology (GO), Kyoto Encyclopedia of Genes and Genomes (KEGG), Reactome, and ImmPort databases, with significance defined as a false discovery rate (FDR) q-value <0.05. Group comparisons used two-tailed unpaired t-tests or Mann–Whitney U tests, data were presented as mean ± SD or standard error of mean. The chi-square test was used to compare binary categorical variables. Correlations were assessed using Spearman’s rank test. For QMG dichotomization (≥6 as positive), logistic regression analyzed ABCs proportion, with area under the curve (AUC) (DeLong’s method), optimal cutoff (Youden index), sensitivity/specificity reported, and receiver operating characteristic (ROC) curve (pROC package) visualized via ggplot2 (R package). Confidence intervals (CI) for the ROC metrics were not further validated using bootstrapping or cross-validation. A *p*-value <0.05 was considered statistically significant.

## 3 Results

### 3.1 Study population and baseline characteristics

This study enrolled 15 HC and 20 naïve patients diagnosed with AChR-MG. The demographic and clinical characteristics of the MG cohort are summarized in Table 1. The immunosuppressant medication history of enrolled MG patients was shown in the Supplementary Figure S2. Comparative analysis revealed no statistically significant differences in sex (assessed by *χ*^2^ test, *p* = 0.70) or median age (evaluated by independent samples *t*-test, *p* = 0.33) between the HC and MG groups (Table 2). These findings indicated balanced baseline characteristics between the cohorts, effectively minimizing the potential confounding effects of demographic variables on subsequent analyses. The homogeneity in baseline profiles underscores the validity of the intergroup comparisons in this study.

**Table 1:** General information of AChR-MG patients.

Patients	Sex	Age	MGFA classification	AChR-Abs titters (nmol/L)	QMG scores
1	Male	46	IIa	2.09	3
2	Female	29	IIa	38.45	5
3	Male	24	IIIa	18.69	10
4	Female	25	IIa	4.96	7
5	Female	45	IIa	6.30	5
6	Female	39	IIb	41.35	9
7	Male	25	IIa	1.23	6
8	Female	24	IIIb	33.73	12
9	Male	50	IIa	1.23	4
10	Female	31	IIb	2.70	7
11	Male	38	IIa	9.38	4
12	Female	49	IIIb	13.79	14
13	Male	35	IIIb	19.23	10
14	Female	44	IIa	7.14	6
15	Female	40	IIa	2.09	5
16	Female	44	IIb	16.47	8
17	Male	48	IIa	10.47	6
18	Male	42	IIIb	23.17	13
19	Female	38	IIa	10.60	4
20	Female	45	IIa	9.17	5

MGFA, Myasthenia Gravis Foundation of America; AChR, acetylcholine receptor; QMG, quantitative myasthenia gravis.

**Table 2:** General information for study participants in two group.

Characteristics	HC	MG	*p* value
Number	15	20	
Age	35.27 (36.00)	38.05 (39.50)	0.33
Sex			
Male	53.33	40.00	0.70
Female	46.67	60.00	0.70

HC, healthy control; MG, myasthenia gravis. The age was showed as median (interquartile range), and the sex was showed as percentage.

### 3.2 Dysregulated B-cell-related pathways in PBMCs of MG

**Figure 1:**
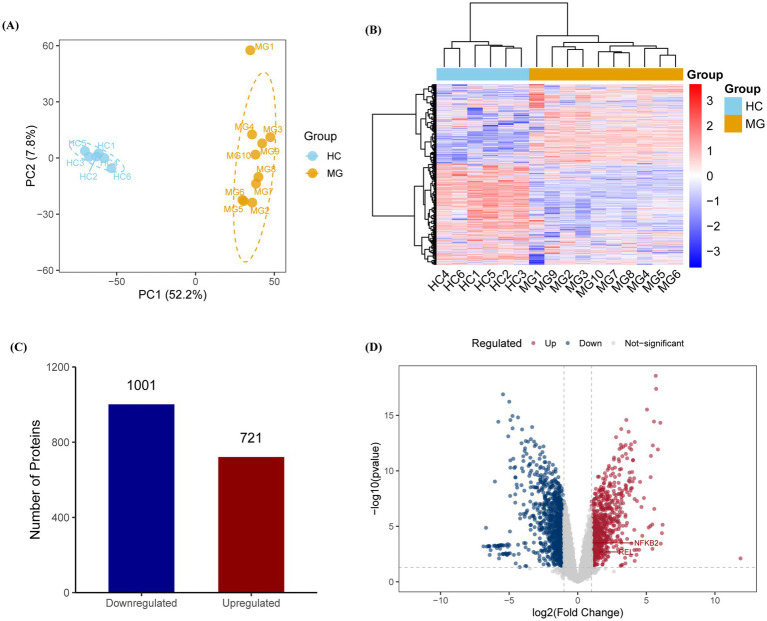
Proteomic profiling reveals distinct molecular signatures in AChR-MG patients. **(A,B)** Principal component analysis (PCA) and hierarchical clustering of PBMC proteomes showing clear separation between MG (*n* = 10) and HC (*n* = 6) groups along principal component 1 (PC1, 52.20% variance) and PC2 (7.80% variance). **(C,D)** Differentially expressed proteins (DEPs) analysis identifies 721 upregulated (red) and 1,001 downregulated (blue) proteins in MG patients. DEPs were identified with thresholds of | log2 (Fold Change) | > 1 and *p*-value <0.05, with multiple testing correction performed via the Benjamini-Hochberg method.

A total of 5,452 proteins were identified using Proteome Discoverer annotations, with 5,167 retained after data normalization. Principal component analysis (PCA) demonstrated that PC1 (52.20%) and PC2 (7.80%) effectively distinguished the MG group from the HC group, highlighting the significant heterogeneity in protein abundance between the two groups (Figures 1A,B). Differential expression analysis identified 721 upregulated and 1,001 downregulated proteins in the MG group compared to those in the HC (Figures 1C,D).

**Figure 2:**
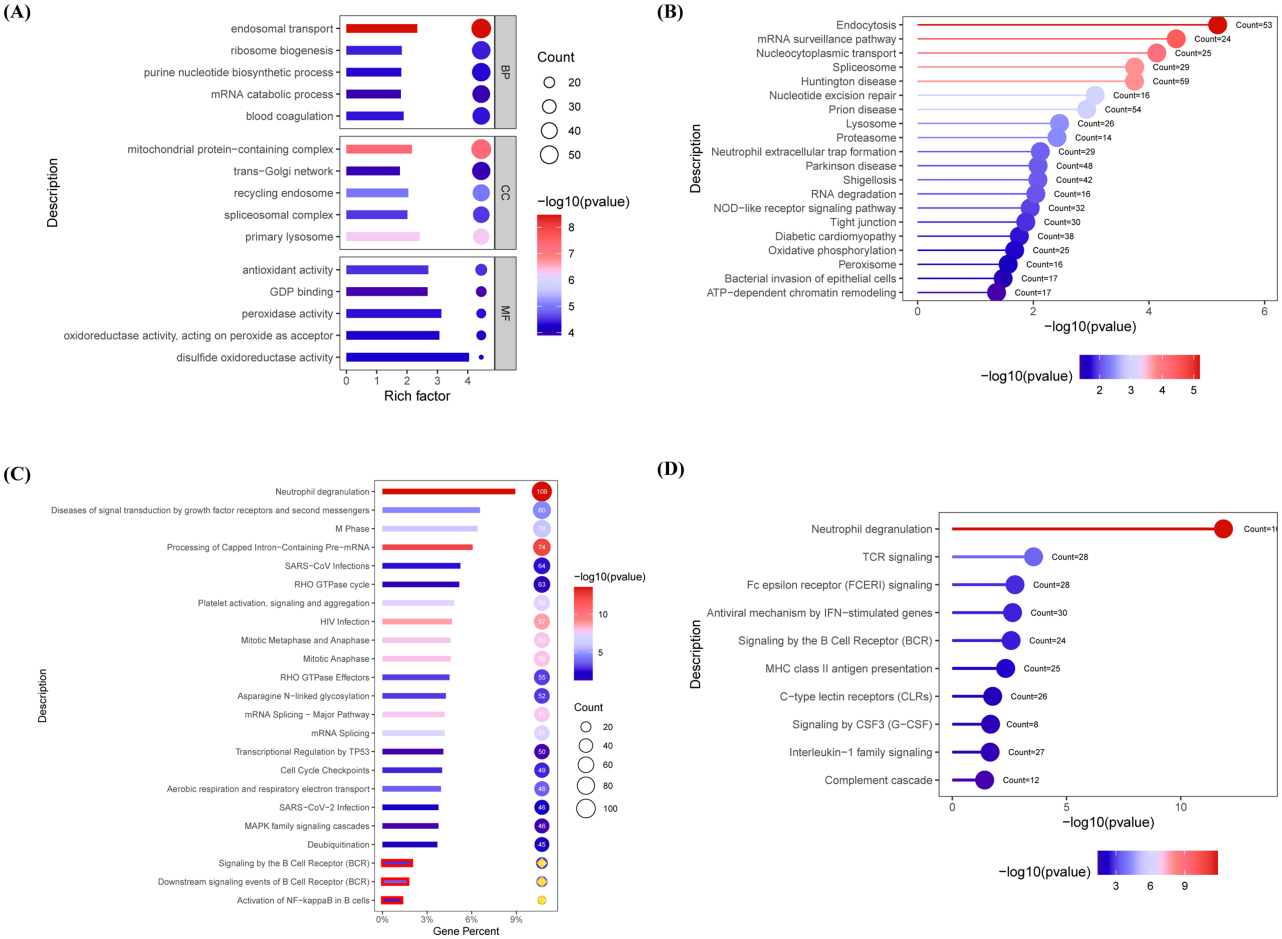
Pathway enrichment analysis reveals B cell receptor signaling and immune dysregulation in AChR-MG. Differentially expressed proteins (DEPs) were functionally annotated through enrichment analyses in the Gene Ontology (GO), Kyoto Encyclopedia of Genes and Genomes (KEGG), Reactome, and ImmPort databases. **(A)** GO enrichment reveals upregulated endosomal transport and mitochondrial protein complexes in MG. **(B)** KEGG analysis highlights endocytosis and mRNA surveillance pathways. **(C)** Reactome pathway annotation demonstrates activation of BCR and NF-κB signaling (*p* < 0.05). **(D)** ImmPort immune pathway enrichment confirms significant involvement of TCR and BCR signaling in MG pathogenesis. Significantly enriched pathways were identified using a false discovery rate (FDR)-adjusted *q*-value threshold of <0.05. Top pathways ranked by gene count and significance; Circle size reflects gene count; Color gradient indicates statistical significance [−log10 (*p* value), blue-to-red: low-to-high].

Functional annotation of DEPs was performed using the GO, KEGG, Reactome, and ImmPort databases, with the top-ranked pathways selected based on gene counts and *p*-value. GO enrichment analysis revealed a significant upregulation of endosomal transport and mitochondrial protein-containing complex pathways in the MG group (Figure 2A). KEGG pathway analysis highlighted enrichment in the endocytosis and mRNA surveillance pathways (Figure 2B). Reactome analysis further identified the activation of B-cell receptor (BCR) and NF-κB signaling pathways in the MG group (Figure 2C). Immune pathway enrichment via ImmPort confirmed the significant involvement of T-cell receptor and BCR signaling pathways in the MG group (Figure 2D).

**Figure 3:**
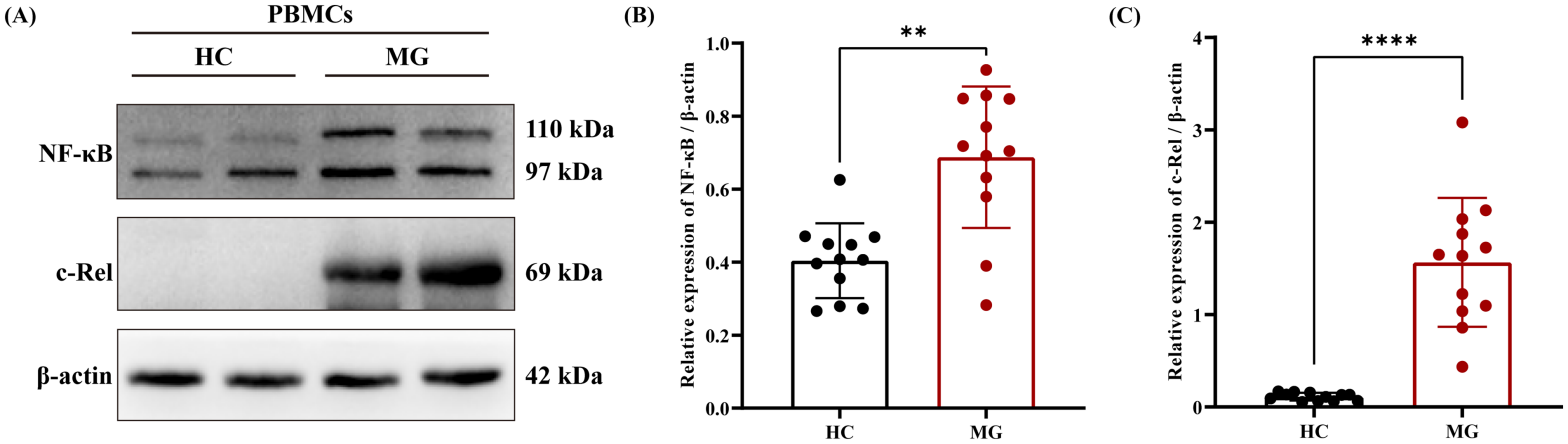
NF-κB/c-Rel signal pathway was upregulated in AChR-MG. Total cellular protein was extracted and detected by western blot. **(A)** Visualization of NF-κB and c-Rel protein expression. **(B,C)** Comparison of the expression levels of NF - κB and c-Rel protein relative to *β*-actin between two groups. Data were expressed as means ± standard deviation (SD), HC (*n* = 12), AChR-MG (*n* = 12).

Given the critical role of the NF-κB signaling pathway in B cell activation and the specific involvement of cellular reticuloendotheliosis (c-Rel), a member of the NF-κB family, in plasma cell differentiation and high-affinity antibody production (20), we further validated the expression levels of the NF-κB/c-Rel signaling pathway in MG patients based on the aforementioned proteomic results using western blot. The results demonstrated that the relative expression levels of NF-κB/c-Rel normalized to *β*-actin were significantly higher in the MG group compared to the HC group (Figures 3A–C).

### 3.3 B-cell subsets imbalance and its association with clinical phenotype

**Figure 4:**
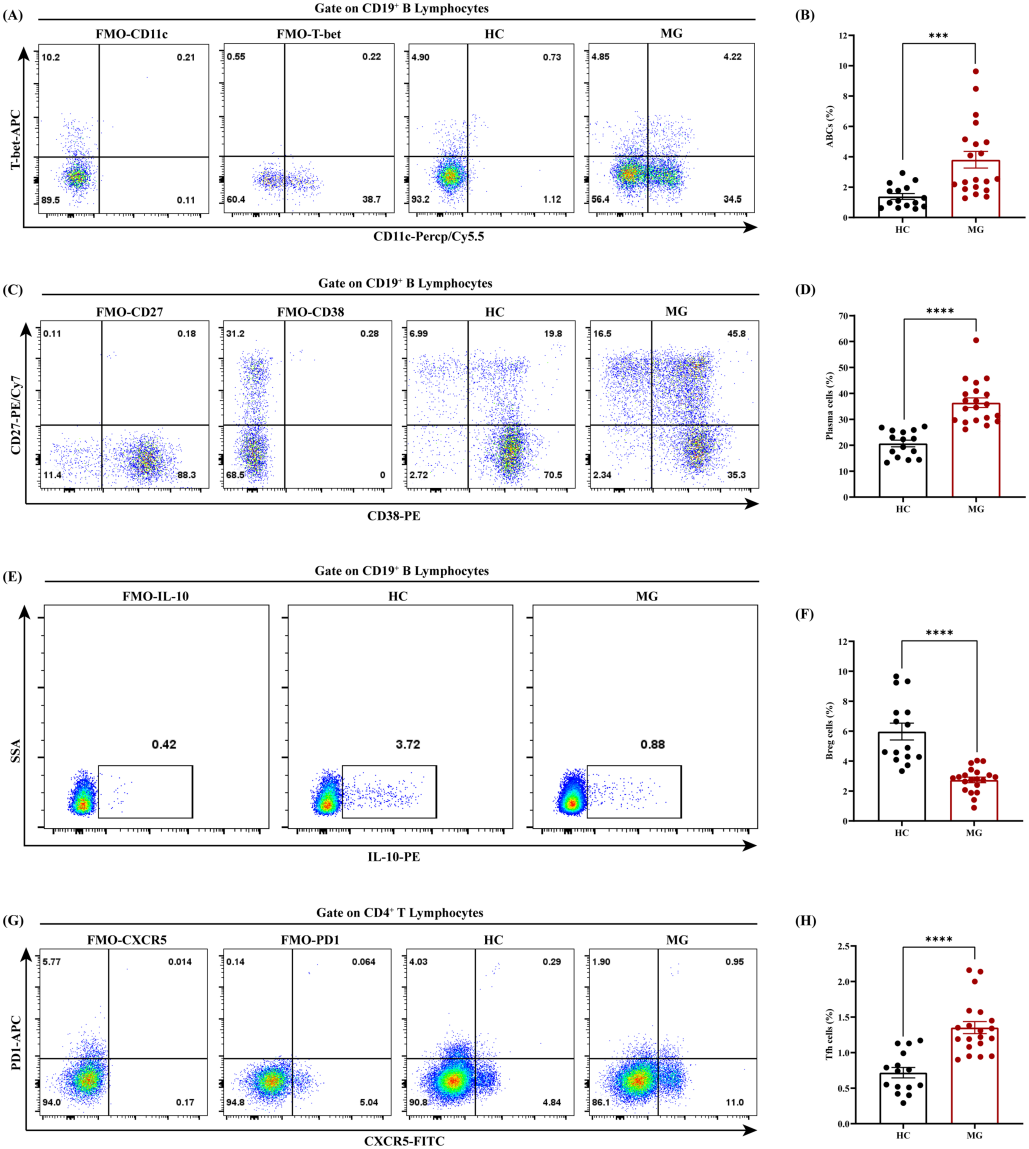
Dysregulated B cell and Tfh subsets in AChR-MG patients. PBMCs from AChR-MG and HC group were analyzed by flow cytometry. **(A,B)** ABCs (CD19^+^CD11c^+^T-bet^+^) are significantly elevated in MG vs. HC (*p* < 0.001). **(C,D)** Plasma cells (CD19^+^CD27^+^CD38^+^) show increased proportions in MG group (***p < 0.001). **(E,F)** Breg cells (CD19^+^IL-10^+^) are markedly reduced in MG group (****p < 0.0001). **(G,H)** Tfh cells (CD4^+^PD1^+^CXCR5^+^) are expanded in MG group (***p < 0.001). Data were expressed as means ± standard error of mean, HC (*n* = 15), AChR-MG (*n* = 20).

**Figure 5:**
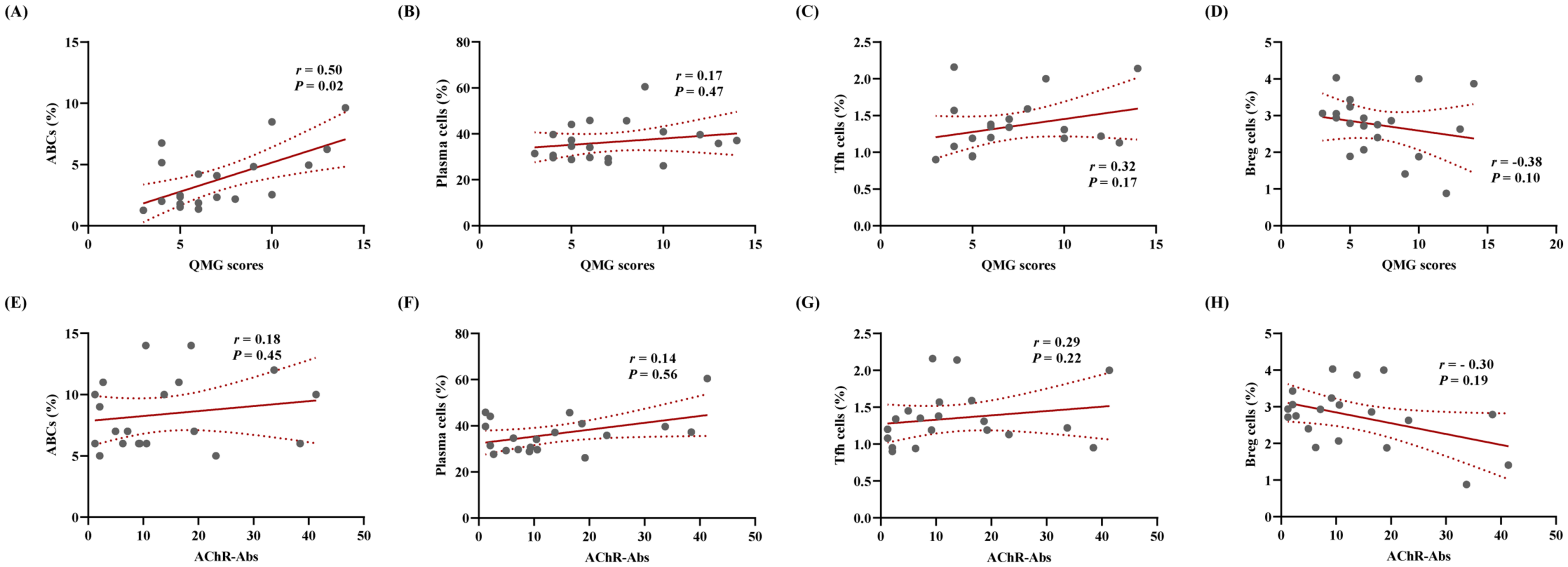
ABCs associate with clinical severity in AChR-MG independent of AChR antibody titers. Correlation analysis was conducted between the proportions of various cell subsets and QMG scores as well as AChR antibody titers. **(A)** Positive association between ABCs and QMG scores (Spearman’s *r* = 0.50, *p* = 0.02). **(B–D)** No significant correlations of QMG scores with Tfh cells, plasma cells, or Breg cells (*p* > 0.05). **(E–H)** Cellular subsets (ABCs, Tfh cells, plasma cells, Breg cells) show no associations with AChR antibody titers (*p* > 0.05; *n* = 20).

Flow cytometry analysis demonstrated that the proportions of ABCs (CD19^+^CD11c^+^T-bet^+^), plasma cells (CD19^+^CD27^+^CD38^+^), and Tfh cells (CD4^+^PD-1^+^CXCR5^+^) in PBMCs were significantly elevated in the MG group compared to HC (*p* < 0.001 for all), whereas regulatory B cells (Bregs) (CD19^+^IL-10^+^) were significantly reduced (*p* < 0.0001; Figures 4A–H). Correlation analyses between these cellular subsets and clinical parameters revealed a positive association between the ABCs and QMG scores (*r* = 0.50, *p* = 0.02; Figure 5A), whereas no significant correlations were observed between QMG scores and other subsets (Tfh, plasma cells, or Bregs; *p* > 0.05; Figures 5B–D). Furthermore, none of the cellular subsets exhibited significant correlations with AChR antibody titers (*p* > 0.05 for all; Figures 5E–H). The raw data used for statistical analysis and effect sizes were shown in the Supplementary Tables S1, S2.

### 3.4 Diagnostic and predictive value analysis

**Figure 6:**
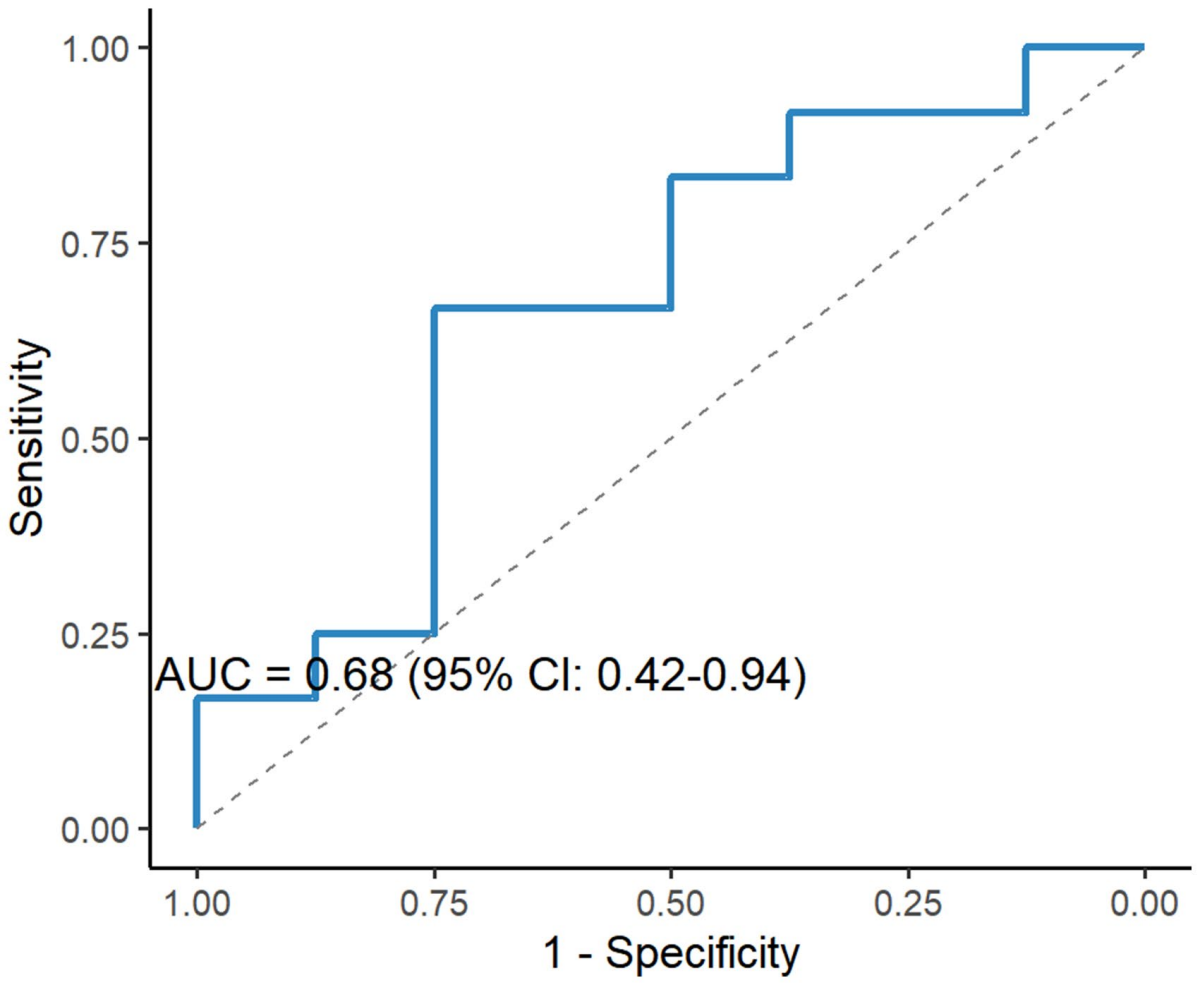
Diagnostic performance of ABCs for moderate-to-severe MG. ROC analysis of ABCs proportions for discriminating moderate-to-severe MG (QMG score ≥ 6), yielding an area under the curve (AUC) of 0.68 (95% CI: 0.42–0.94), sensitivity = 65%, specificity = 72%. Dashed line indicates reference (AUC = 0.5).

To analyze the diagnostic and predictive value of ABCs in MG, ROC curve analysis was conducted to assess the diagnostic value of ABCs proportion for moderate-to-severe MG (QMG scores ≥ 6), revealing an AUC of 0.68 (95% CI: 0.42–0.94) with sensitivity and specificity of 65 and 72%, respectively (Figure 6). Although the AUC indicated moderate discriminative capacity, ABCs may provide complementary diagnostic value when combined with other biomarkers.

## 4 Discussion

MG is an acquired autoimmune disorder associated with cell-mediated immunity, which depends on humoral immunity, and involves complement activation. Abnormal secretion of autoantibodies by B cells is a critical factor in the dysregulation of humoral immunity, in which naïve B cells differentiate into functionally diverse subsets in response to varying antigen stimuli. An increasing number of B-cell-targeted therapies have been applied in clinical practice for MG, demonstrating certain benefits (27). However, CD20-targeted B-cell depletion therapies, which broadly suppress humoral immunity, predispose patients to severe infections, and a subset of individual’s exhibit poor or no response to current therapies (28). Therefore, an in-depth analysis of B cell subsets and activation pathways, along with the identification of novel biomarkers targeting specific B cell populations, may advance precision therapy for MG. This study employed proteomics and phenotypic profiling to comprehensively reveal dysregulated B cell associated pathways and subsets in the pathogenesis of AChR-MG. The findings focused on three core dimensions: (1) proteomic abnormalities in B cell activation pathways, (2) imbalance between pathogenic and regulatory B cell subsets, and (3) the association of ABCs with clinical phenotypes, highlighting novel biomarkers and therapeutic targets.

B cell immune dysregulation plays a pivotal role in the pathogenesis of MG. Aberrantly activated B cells disrupt neuromuscular junction signaling by secreting autoantibodies against self-antigens (27, 29). As antigen-presenting cells, B cells present NMJ antigens to CD4^+^ T cells via MHC-II molecules, driving Th1/Th17 and Tfh cell differentiation, further amplifying B cell activation and antibody class switching (30). The heterogeneous differentiation of B-cell subsets in MG underscores the importance of B-cell-targeted therapies, which have emerged as key treatment strategies. Previous studies indicate that the NF-κB signaling pathway contributes to immune dysregulation in MG by activating B cells through BCR or B cell activating factor pathways (25, 31). In this study, proteomic profiling revealed significant activation of the BCR and NF-κB/c-Rel signaling pathways in MG patients, a finding validated by upregulated NF-κB/c-Rel expression via western blot analysis. These results align with prior research, reinforcing central role of NF-κB in regulating B cell differentiation and autoantibody production in MG (19, 20, 25). Furthermore, proteomic enrichment of endocytosis and mRNA surveillance pathways suggested that enhanced antigen presentation and post-transcriptional regulation in MG B cells may exacerbate autoimmune responses. Aberrant activation of NF-κB represents a critical mechanism underlying B cell pathogenicity in MG and other autoimmune diseases. Although these molecules represent mechanistically plausible therapeutic targets, their translational potential requires validation in preclinical models and clinical trials that directly assess the pathway modulation in MG.

An imbalance in B cell subsets is a central driver of abnormal humoral immune responses in MG, characterized by increased pro-inflammatory B cells and reduced immunosuppressive B cell populations (32). However, studies have suggested differences in the B cell subsets distribution between MG subtypes (ocular and gMG), underscoring the high heterogeneity of MG as an autoimmune disorder (33). In this study, MG patients exhibited significantly elevated proportions of ABCs, plasma cells, and Tfh cells in the peripheral blood, along with decreased Bregs, indicating a pro-inflammatory B cell microenvironment in MG. These findings align with previous reports (32, 33). Notably, ABCs showed a positive correlation with QMG scores (*r* = 0.50) but no significant association with AChR antibody titers. These observations raise the hypothesis that ABCs may contribute to the modulation of disease severity through antibody-independent mechanisms in MG. Based on their established capacity to secrete inflammatory cytokines such as IFN-*γ* (13, 34), we propose that potential mechanisms could include IFN-γ secretion or T cell co-stimulation; however, this remains speculative and requires future functional validation in the context of MG. In contrast, the lack of a correlation between Bregs and QMG scores may reflect insufficient compensatory capacity during disease progression. In parallel with established MG biomarkers like pathogenic Th17 cells and Treg cells which showed correlations with disease severity in our prior studies (26, 35), ABCs add unique value through their direct mechanistic link to pathogenic B cell hyperactivity.

ABCs represent a distinct B-cell subset, potentially differentiating independently from BCR pathway stimulation while retaining antibody-secreting functionality (36). It is well documented that in autoimmune diseases, ABCs can exhibit unique cytokine profiles distinct from other B cells, including the production of pro-inflammatory mediators like IL-6, IFN-*γ*, and TNF-*α* (13, 34). Studies in other contexts have proposed that bidirectional interactions between ABCs and Tfh cells, mediated by IL-21 and IFN-γ signaling, may drive ABC differentiation (37, 38). While our study, which focused on correlative relationships, did not find a statistically significant association between ABCs and Tfh cells (*r* = 0.41, *p* = 0.08), a trend suggesting a positive relationship was observed. This observed trend, albeit non-significant and potentially due to the sample size, raises the interesting hypothesis that similar interaction pathways might also operate in MG, further motivating future mechanistic investigations. These results have been included in the Supplementary Figure S3.

The identification of novel biomarkers contributes to a better understanding of the pathological mechanisms of MG and enables precise and effective clinical therapies (39). Existing studies have confirmed that abnormal differentiation of ABCs correlates with clinical disease activity in multiple autoimmune disorders (8, 11, 12). Our findings demonstrated an association between ABCs and QMG scores, suggesting shared disease mechanisms. Although ABCs’ biomarker potential in MG was previously unexplored, our ROC analysis reveals moderate diagnostic accuracy for moderate-to-severe MG (QMG scores ≥ 6; AUC = 0.68, 95% CI: 0.42–0.94). While a wide CI indicates sample size limitations requiring multi-center validation, combining ABCs with conventional biomarkers (AChR antibodies and MGFA classification) could enhance diagnostic precision. Given their clinical phenotype correlation, we propose that ABCs may serve as complementary biomarkers with three potential applications: (1) therapy intensity guidance for high-ABC subgroups; (2) enrichment of clinical trial populations; and (3) monitoring disease activity beyond antibody titers. Future studies should prioritize the longitudinal assessment of ABC dynamics during therapy (e.g., B-cell depletion) using standardized clinical endpoints to evaluate their utility as response indicators.

## 5 Conclusion

This study, through multidimensional analysis, highlights the central role of ABCs and the NF-κB/c-Rel signaling pathway in AChR-MG. These findings provide a theoretical foundation for the development of precise immunotherapies that target pathogenic B cell subsets and their upstream signaling nodes. The limitations of this study include: (1) the cross-sectional design, which precludes causal inferences between B-cell subsets and disease progression; (2) this proteomic analysis requires future validation in larger cohorts given its limited sample size; (3) a relatively small sample size, potentially limiting the power of subgroup analyses; and (4) the absence of functional experiments (e.g., cytokine profiling) to further elucidate the underlying mechanisms. Because this was a pilot exploratory study with a limited sample size, our findings need to be validated in larger, multi-center cohorts in future researches. In addition, future studies could integrate single-cell transcriptomics to delineate B cell heterogeneity and validate the therapeutic potential of NF-κB/c-Rel as a target using preclinical models.

## Data Availability

The mass spectrometry proteomics data have been deposited to the ProteomeXchange Consortium (https://proteomecentral.proteomexchange.org) via the iProX partner repository with the dataset identifier PXD067024. The other raw data supporting the conclusions of this article will be made available by the authors, without undue reservation.
