# Supplementary material for: CD19+CD11c+T-bet+ B cells in myasthenia gravis: a potential biomarker
Source: Front Neurol. 2025 Aug 22;16:1623066. doi: 10.3389/fneur.2025.1623066 (PMC12411748; doi:10.3389/fneur.2025.1623066)
Supplement: Supplementary file 1 [file Data_Sheet_1.docx]

**Table S1 The raw data used for statistical analysis and effect sizes in Figures 3 and 4**

|  | HC  (Mean ± SD) | MG  (Mean ± SD) | *P* value | *d* estimate | 95 % CI |
| --- | --- | --- | --- | --- | --- |
| NF-κB/β-actin | 0.40 ± 0.10 | 0.69 ± 0.19 | 0.0011 | 1.83 | 0.82 ~ 2.84 |
| c-Rel/β-actin | 0.11 ± 0.04 | 1.57 ± 0.70 | < 0.0001 | 2.94 | 1.72 ~ 4.16 |
| ABCs (%) | 1.39 ± 0.76 | 3.81 ± 2.45 | 0.0008 | 1.26 | 0.50 ~ 2.02 |
| Plasma cells (%) | 20.69 ± 4.97 | 36.41 ± 8.30 | < 0.0001 | 2.22 | 1.34 ~ 3.10 |
| Breg cells (%) | 5.97 ± 2.17 | 2.74 ± 0.82 | < 0.0001 | -2.09 | -2.96 ~ -1.23 |
| Tfh cells(%) | 0.72 ± 0.29 | 1.35 ± 0.38 | < 0.0001 | 1.85 | 1.02 ~ 2.68 |

HC: healthy control; MG: myasthenia gravis; CI: confidence intervals; NF-κB: nuclear factor kappa-B; ABCs: age-associated B cells; Breg: regulatory B cell; Tfh cell: T follicular helper cell.

**Table S2 The effect sizes of correlation analysis in Figure 5**

|  | *r* | 95% CI | *P* value | Sample number |
| --- | --- | --- | --- | --- |
| QMGs vs ABCs | 0.50 | 0.06 ~ 0.78 | 0.024 | 20 |
| QMGs vs Tfh cells | 0.32 | -0.16 ~ 0.68 | 0.169 | 20 |
| QMGs vs Breg | -0.38 | -0.71 ~ 0.09 | 0.103 | 20 |
| QMGs vs Plasma cells | 0.17 | -0.31 ~ 0.58 | 0.474 | 20 |
| AChR-Abs vs ABCs | 0.18 | -0.30 ~ 0.59 | 0.450 | 20 |
| AChR-Abs vs Tfh cells | 0.29 | -0.19 ~ 0.66 | 0.217 | 20 |
| AChR-Abs vs Breg | -0.30 | -0.67 ~ 0.17 | 0.194 | 20 |

QMGs: quantitative myasthenia gravis scores; CI: confidence intervals; ABCs: age-associated B cells. AChR-Abs: anti-acetylcholine receptor antibodies, Breg: regulatory B cell; Tfh cell: T follicular helper cell.
