# Supplementary figures and images for: CD19+CD11c+T-bet+ B cells in myasthenia gravis: a potential biomarker
Source: Front Neurol. 2025 Aug 22;16:1623066. doi: 10.3389/fneur.2025.1623066 (PMC12411748; doi:10.3389/fneur.2025.1623066)

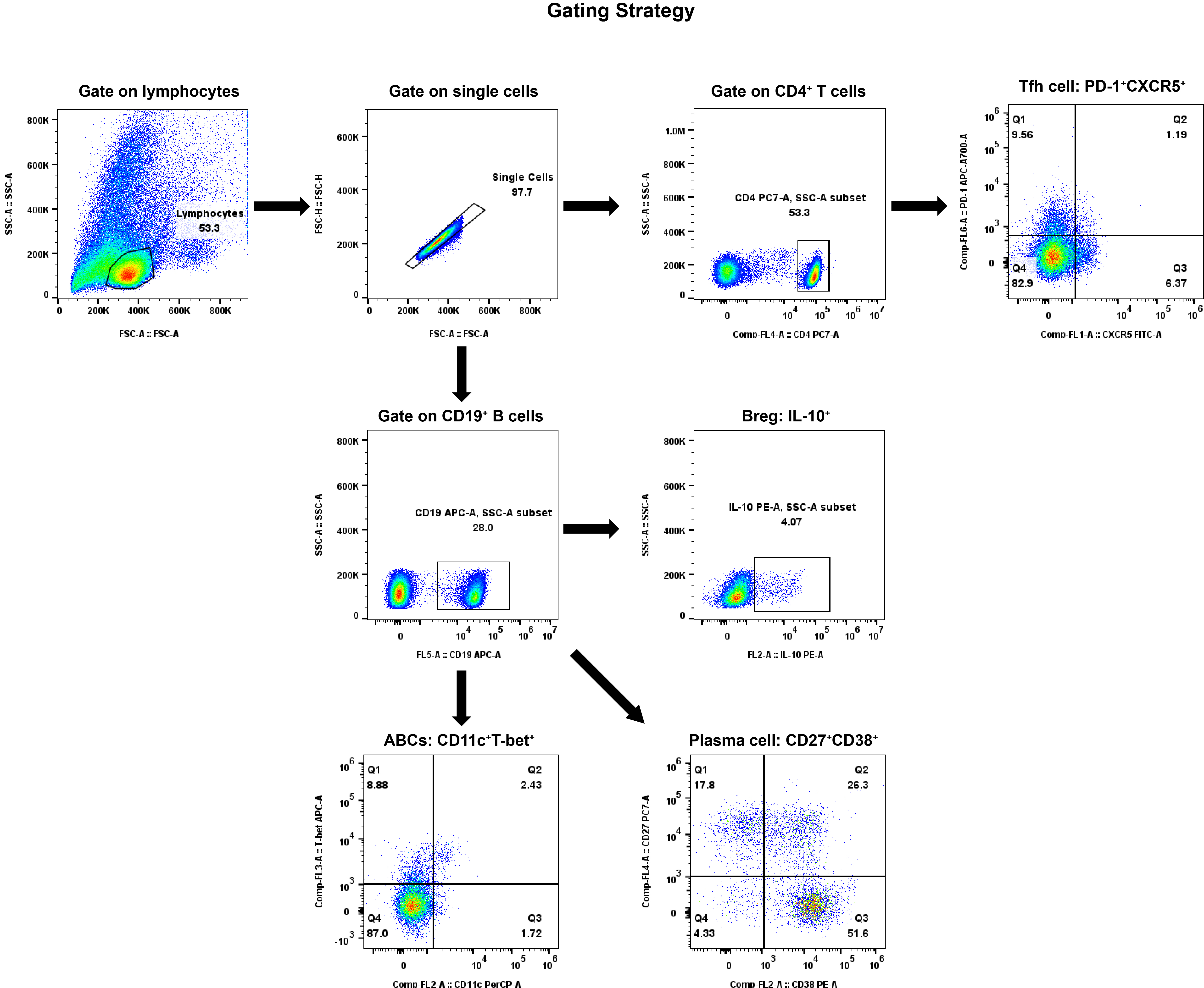

Supplement: Supplementary file 2 [file Image_1.jpg]

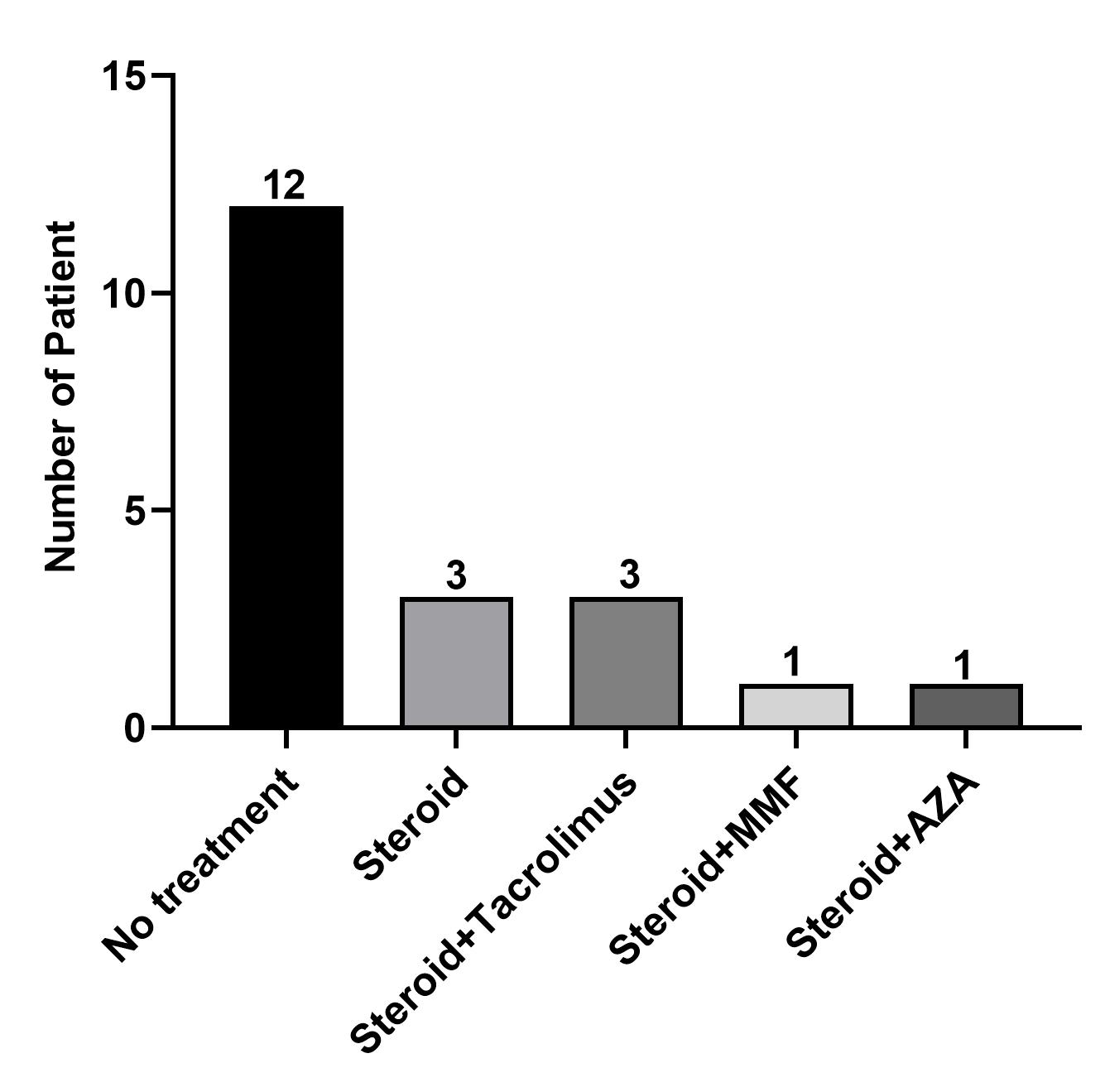

Supplement: Supplementary file 3 [file Image_2.jpg]

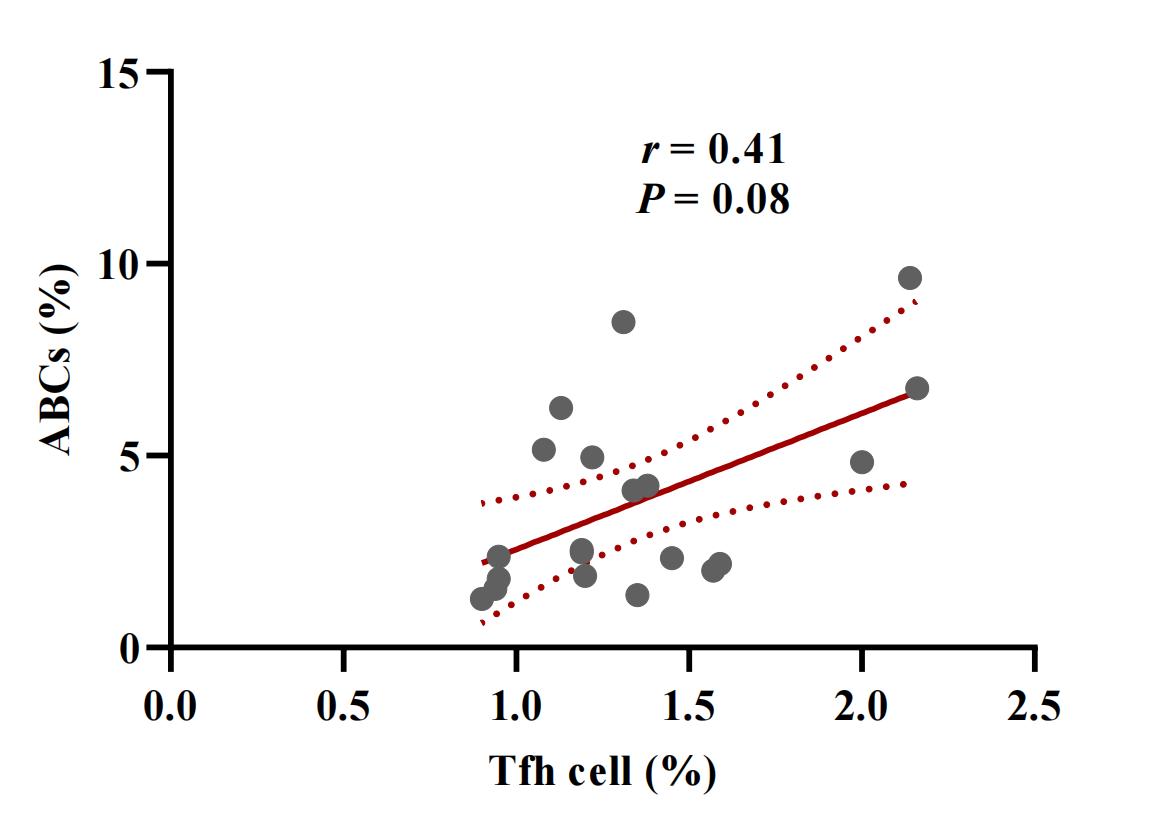

Supplement: Supplementary file 4 [file Image_3.jpg]
